# A comparison of the stem cell characteristics of murine tenocytes and tendon-derived stem cells

**DOI:** 10.1186/s12891-018-2038-2

**Published:** 2018-04-12

**Authors:** Katie Joanna Lee, Peter David Clegg, Eithne Josephine Comerford, Elizabeth Gail Canty-Laird

**Affiliations:** 1 Department of Musculoskeletal Biology, Institute of Ageing and Chronic Disease, University of Liverpool, William Henry Duncan Building, 6 West Derby Street, Liverpool, L7 8TX UK; 2 School of Veterinary Science, Leahurst Campus, University of Liverpoo, Chester High Road, Neston, CH64 7TE UK; 3 The MRC-Arthritis Research UK Centre for Integrated research into Musculoskeletal Ageing (CIMA), Liverpool, UK

**Keywords:** Tendon, Tendon-derived stem cell, Tenocyte, Murine

## Abstract

Tendon is a commonly injured soft musculoskeletal tissue, however, poor healing potential and ineffective treatment strategies result in persistent injuries and tissue that is unable to perform its normal physiological function. The identification of a stem cell population within tendon tissue holds therapeutic potential for treatment of tendon injuries. This study aimed, for the first time, to characterise and compare tenocyte and tendon-derived stem cell (TDSC) populations in murine tendon. Tenocytes and TDSCs were isolated from murine tail tendon. The cells were characterised for morphology, clonogenicity, proliferation, stem cell and tenogenic marker expression and multipotency. TDSCs demonstrated a rounded morphology, compared with a more fibroblastic morphology for tenocytes. Tenocytes had greater clonogenic potential and a smaller population doubling time compared with TDSCs. Stem cell and early tenogenic markers were more highly expressed in TDSCs, whereas late tenogenic markers were more highly expressed in tenocytes. Multipotency was increased in TDSCs with the presence of adipogenic differentiation which was absent in tenocytes. The differences in morphology, clonogenicity, stem cell marker expression and multipotency observed between tenocytes and TDSCs indicate that at least two cell populations are present in murine tail tendon. Determination of the most effective cell population for tendon repair is required in future studies, which in turn may aid in tendon repair strategies.

## Background

Tendon is prone to injury and degeneration, and this is most often seen in occupational and sporting environments [1–3]. The healing process for tendon is poorly understood, however it is well documented that tendon tissue is unable to heal effectively resulting in painful and debilitating scar tissue, which is unable to perform its normal physiological function [1, 4]. The current treatment options for damaged or degenerated tendon vary depending on the severity and location of the tendinopathy [5–8] and include physiotherapy; pharmacotherapies, such as anti-inflammatories; corticosteroid injections; or surgery [5, 6, 9]. However, these treatment strategies are largely ineffective [5]; therefore, an alternative approach for the management and treatment of tendinopathies is currently being sought.

Tenocytes are tendon-specific fibroblasts and traditionally were thought to be the only cell type present in tendon, however it is now thought that tenocytes account for approximately 95% of the cellular content of tendon, with progenitor cells, endothelial cells and chondrocytes comprising the remaining 5% [10]. Tenocytes are located between collagen fibrils and in the interfascicular matrix and they are responsible for the production of the ECM as well as the repair and maintenance of tendon tissue [10, 11]. The identification of a stem cell population within tendon tissue [12] holds therapeutic potential for treatment of tendon injuries. Tendon-derived stem cells (TDSCs) have been shown to be clonogenic, multipotent and express stem cell and tenogenic markers [12–15].

A number of tissue engineering strategies have utilised TDSCs for tendon repair with some successful outcomes [16–20]. These studies highlight the potential use of TDSCs in tendon repair strategies, however further characterisation of TDSCs is necessary; particularly, the identification and characterisation of different cell populations within tendon tissue. Comparisons of tendon cell populations are lacking in the literature with only two studies comparing tenocytes and TDSC properties in the rabbit [14] and the horse [15]. These two studies reported conflicting results with large differences found between tenocyte and TDSC populations in the rabbit [14], but few differences observed in the horse [15]. No studies, to date, have compared tendon cell populations in rodents, despite the plethora of research on TDSCs in rats and mice.

This study aimed to isolate, characterise and compare tenocytes and TDSCs from murine tail tendon. We hypothesised that tenocytes would demonstrate phenotypic differences when compared with TDSCs, particularly differences in stem cell properties.

## Methods

### Isolation of murine tenocytes and TDSCs

HuR floxed embryos were obtained from Dimitris Kontoyiannis, Alexander Fleming Research Centre, Greece [21] and crossed with Aggrecan A1 Cre mice obtained from George Bou-Gharios, University of Liverpool, UK [22]. Tendon tissue was extracted from the tails of 6–8 week old C57BL/6 mice (HuR^fl/fl^*Acan*-Cre^+/−^) which were euthanased for reasons unrelated to this study, and digested for 3 h at 37 °C in 20 ml 375 U/ml collagenase type I and 0.05% trypsin. The resulting cell suspension was strained and then centrifuged at 1200 g for 10 min and the supernatant discarded. The cells were resuspended in complete DMEM (DMEM supplemented with 20% foetal calf serum, 100 U/ml penicillin, 100 μg/ml streptomycin and 2 μg/ml amphotericin B) and counted using a haemocytometer. For tenocyte isolation the cells were seeded at 1 × 10^5^ cells in T25 culture flasks (4 × 10^3^ cells/cm^2^) [23, 24] and for TDSC isolation the cells were seeded at 100 cells per well of a 6-well plate (10 cells/cm^2^) [13, 15, 16, 25–28]. All cells were cultured in complete DMEM at 37 °C, 5% CO_2_ and 21% O_2_. TDSCs were cultured for 6–8 days before passaging, whereas tenocytes were cultured for 2–3 days, cells were split 2:1 for subsequent passages. For TDSCs colonies were isolated using cloning cylinders and local application of 0.05% trypsin. All cells were analysed at passage 2–3 [15].

### Cell proliferation assay

Cells at passage 2 were seeded at 10,000 cells in T25 culture flasks at day 0. At 80% confluency the cells were counted and the doubling time calculated using the formula below:

(LOG_10_(cell number after proliferation)-LOG_10_(initial seeding density))/LOG_10_(2) [29].

### Colony formation assay

Cells at passage 2 were seeded at 100 cells/cm^2^ in 6-well cell culture plates. After 7 days in culture the cells were washed and then fixed with 6% gluteraldehyde and stained with 0.5% crystal violet solution [30]. The cells were washed again and imaged using a biomolecular imager (Typhoon FLA 7000, GE Healthcare) and analysed using ImageQuant software (GE Healthcare) for colony number and size.

### Tri-lineage differentiation assays

Cell monolayers were cultured for 21 days in osteogenic (complete DMEM containing 100 nM dexamethasone, 10 mM β-glycerophosphate and 50 mM ascorbic acid) [31] and adipogenic (complete DMEM containing 1 μM dexamethasone, 100 μM indomethacin, 10 μg/ml insulin and 500 μM IBMX) [32] induction media. Cell pellets (containing 5 × 10^5^ cells) were cultured for 21 days in chondrogenic (complete DMEM containing 100 nM dexamethasone, 25 μg/ml ascorbic acid, 10 ng/ml TGF-β3 and ITS+ 3 supplement) [33] induction media. Control cells for all treatments were cultured in complete DMEM. After culturing, the cells were stained with alizarin red and alkaline phosphatase to assess osteogenic differentiation, Oil Red O to assess adipogenic differentiation, or alcian blue for chondrogenic differentiation, as described in the PromoCell MSC application notes (http://www.promocell.com/downloads/application-notes/). Chondrogenic pellets were also paraffin embedded and 4 μm sections taken which were rehydrated and further stained with 1% Alcian blue solution and 0.1% Safranin O solution. In addition, separate cell pellets were digested in 10 U/ml papain solution for 3 h at 60 °C before the total sulphated glycosaminoglycan (sGAG) content was quantified. Dimethylmethylene blue dye was added to each sample and the absorbance read immediately at 570 nm. The sGAG content was calculated from a standard curve produced using chondroitin sulphate standards [34]. RNA was extracted from all assays to analyse lineage-specific gene expression.

### RNA extraction and quantitative real time-polymerase chain reaction (qRT-PCR)

RNA was extracted from all cell types by firstly applying Trizol to cell monolayers and using a cell scraper for cell detachment. After vortexing and centrifugation, 50 μg/ml glycoblue and 100% isopropanol were added to the aqueous phase for RNA precipitation. After centrifugation, the pellets were washed in 75% ethanol and resuspended in Tris-EDTA buffer. The quantity and quality of RNA was assessed using a NanoDrop spectrophotometer (Thermo Fisher). 4 U DNase was then added to the samples to remove DNA, after which time an equal volume of phenol:chloroform:IAA was added to each sample. The RNA was then precipitated, centrifuged, washed in ethanol and the RNA quality assessed. cDNA was synthesised in a 25 μl reaction from 1 to 2 μg of total RNA. The conditions for cDNA synthesis were: incubation at 5 min at 70 °C, 60 min at 37 °C and 5 min at 93 °C with M-MLV reverse transcriptase and random-hexamer oligonucleotides (Promega) [35, 36].

qRT-PCR was conducted using a GoTaq(R) qPCR Master Mix (Promega), and in a 25 μl reaction 10 ng of cDNA was amplified in an AB 7300 Real Time PCR System (Applied Biosystems). After an initial denaturation for 10 min at 95 °C, 40 PCR cycles were performed consisting of 15 s at 95 °C and 1 min at 60 °C. Relative gene expression was calculated according to the comparative C_t_ method [35–37]. Murine specific primers were used (Table 1) and GAPDH was used as an internal control. Primers were designed using Primer-BLAST (NCBI), and the quality of each primer was tested using NetPrimer (Premier Biosoft). In addition, each primer was subjected to a BLAST (NCBI) search to ensure specificity. The best housekeeping gene was determined using the geNorm algorithm [38] and all primers were tested for efficiency; efficiencies between 90 and 110% were deemed to be acceptable.

**Table 1:** Primer sequences for murine genes

Gene	Forward Primer	Reverse Primer
GAPDH	GAGAGGCCCTATCCCAACTC	GTGGGTGCAGCGAACTTTAT
CD90	GGATGAGGGCGACTACTTTTGT	TTGGAGCTCATGGGATTCG
CD73	TGGTTCACCGTTTACAAAGG	CGCTCAGAATTGGAAATTTAAC
TNC	AGGCGATCCCAGCCAGTCAGT	ATGGACGGGGCACCTCCTGTC
SCX	AAGTTGAGCAAAGACCGTGACA	TGTGGACCCTCCTCCTTCTAAC
MKX	AGTAAAGACAGTCAAGCTGCCACTG	TCCTGGCCACTCTAGAAGCG
Sca-1	GTTTGCTGATTCTTCTTGTGGCCC	ACTGCTGCCTCCTGAGTAACAC
NANOG	AGGGTCTGCTACTGAGATGCTCTG	CAACCACTGGTTTTTCTGCCACCG
TNMD	AACTCCACCTCAGCAGTAGTCC	TTTCTTGGATACCTCGGGCCAGAA
THBS4	TCCTCCGCTACCTGAAGAATGATGG	TTCAATGGACTCTGGGTTCTGGGTG
CD45	AGTTAGTGAATGGAGACCAGGAA	TCCATAAGTCTGCTTTCCTTCG
RUNX2	ATGCGTATTCCTGTAGATCCG	TTGGGGAGGATTTGTGAAGAC
OC	CTCTGTCTCTCTGACCTCACA	CAGGTCCTAAA AGTGATACC
OSX	GAAAGGAGGCACAAAGAAG	CACCAAGGAGTAGGTGTGTT
OPN	CATGAGATTGGCAGTGATTTGC	TGCAGGCTGTAAAGCTTCTCCT
FABP4	GAAGCTTGTCTCCAGTCAAAA	AGTCACGCCTTTCATAACACAT
PPARγ	CTCCGTGATGGAAGACCACTC	AGACTCGGAACTCAATGGC
LEPTIN	CTTCACCCCATTCTGAGTTTGT	TTCTCCAGGTCATTGGCTATCT
SOX9	TGGCAGACCAGTACCCGCATCT	TCTTTCTTGTGCTGCACGCGC
COL2A1	GGTTTGGAGAGACCATGAAC	TGGGTTCGCAATGGATTGTG
AGG	TTGCCAGGGGGAGTTGTATTC	GACAGTTCTCACGCCAGGTTTG

### Statistical analysis

Statistical analysis was performed using SPSS (IBM) and SigmaPlot (Systat Software Inc). To ensure data was normally distributed Shapiro Wilk tests were performed. For normally distributed data parametric tests were used for pairwise comparisons. For data which was not normally distributed Log_10_ data transformations were performed resulting in normally distributed data. For pairwise comparisons paired or independent Student’s t-tests were used. *P*-values ≤0.05 were taken to be significant.

## Results

### Tenocyte and TDSC morphology and colony formation

**Fig. 1:**
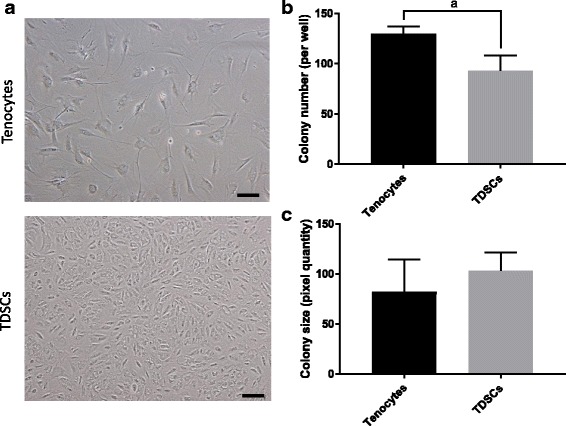
Tenocyte and TDSC morphology and colony formation. Representative images of cell morphology are shown, bars = 100 μm (**a**). Colonies were counted (**b**) and measured (**c**) using ImageQuantTL software. Error bars shown represent SD. Pairwise comparisons were performed using a Student’s independent t-test. ^a^*p* = 0.01. *n* = 4 biological replicates

Tenocytes and TDSCs demonstrated varying cell morphologies; tenocytes were large, flat and fibroblastic, whereas TDSCs were smaller and more rounded (Fig. 1a).

Both cell types were able to form colonies, however these colonies were not homogeneous. Tenocytes generally formed large sparse colonies, whereas TDSCs formed more compact, dense colonies. When quantified tenocytes produced significantly more colonies than TDSCs (Fig. 1b), however colony size was similar between cell types (Fig. 1c).

### Tenocyte and TDSC proliferation

**Fig. 2:**
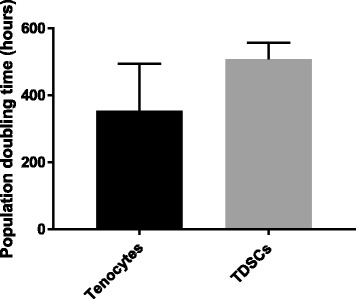
Population doubling time for tenocytes and TDSCs. Error bars shown represent SD. Pairwise comparisons were performed using a Student’s independent t-test. *n* = 4 biological replicates

Both tenocytes and TDSCs proliferated very slowly and demonstrated very long population doubling times (PDT) with a mean (± SD) of 354 (±140) and 508 (±49) hours respectively (Fig. 2).

### Tenocyte and TDSC marker expression

**Fig. 3:**
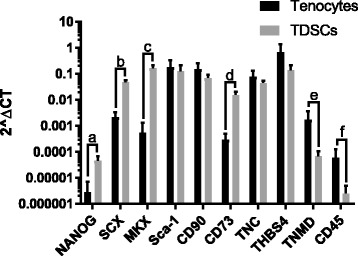
Gene expression analysis of stem cell markers in tenocytes and TDSCs. Values are shown on a logarithmic scale and normalised to GAPDH. Error bars shown represent SD. Pairwise comparisons were performed using independent Student’s t-tests after Log_10_ transformation of data. ^a^*p* = 0.009, ^b^*p* = 0.011, ^c^*p* = 0.001, ^d^*p* = 0.011, ^e^*p* = 0.011, ^f^*p* = 0.011. *n* = 6 biological replicates

The gene expression of stem cell and tenogenic markers was assessed by qRT-PCR (Fig. 3). The majority of stem cell (Nanog and CD73) and early tenogenic markers (scleraxis and Mohawk) were more highly expressed in TDSCs when compared with tenocytes, whereas markers found in developed tendon (tenascin C, thrombospondin-4 and tenomodulin) exhibited higher expression in tenocytes compared to TDSCs. Expression of Nanog, scleraxis and Mohawk was significantly increased in TDSCs compared with tenocytes. Tenomodulin expression was significantly increased in tenocytes compared with TDSCs. The stem cell markers Sca-1 and CD90 were similarly expressed in both cell types. The haematopoietic stem cell marker CD45 demonstrated low expression with significantly higher levels observed for tenocytes compared with TDSCs.

### Tenocyte and TDSC tri-lineage differentiation capacity

The ability of tenocytes and TDSCs to differentiate into different cell lineages was analysed by staining, glycosaminoglycan (GAG) assays and qRT-PCR for gene expression analysis.

**Fig. 4:**
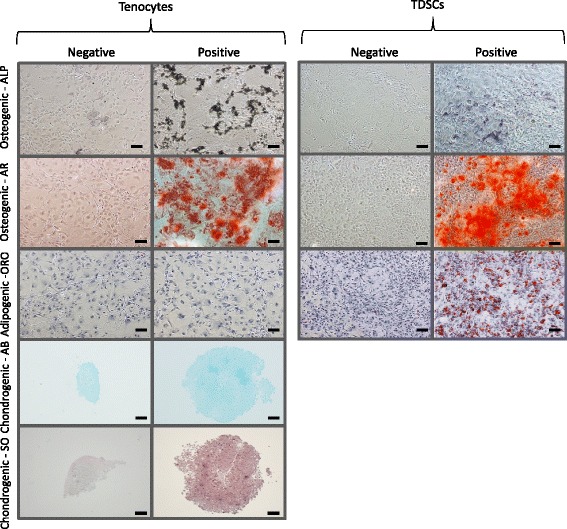
Histological analysis of tri-lineage differentiation potential of tenocytes and TDSCs. Representative images are shown for both cell types after induction of osteogenic, adipogenic and chondrogenic differentiation (positive) and also for control samples (negative), after appropriate staining. Cells subjected to osteogenic differentiation media were stained for both alkaline phosphatase (ALP) activity and calcium deposits using alizarin red (AR). Cells subjected to adipogenic differentiation media were stained for oil droplet formation using oil red O (ORO), and cell pellets exposed to chondrogenic differentiation media, for GAG formation using alcian blue (AB) and safranin O (SO). Bar = 100 μm. Chondrogenic staining was not performed on TDSCs due to low cell numbers. *n* = 6 biological replicates

Both cell types demonstrated osteogenic differentiation as assessed by alkaline phosphatase levels and alizarin red staining (Fig. 4). No adipogenic differentiation was observed for tenocytes, however oil red O staining was seen in differentiated TDSCs (Fig. 4). Tenocytes demonstrated some chondrogenic differentiation, with an increase in pellet size and intensity of safranin O staining in positive samples (chondrogenic induction media) compared with negative samples (control media). Due to low cell numbers, it was not possible to undertake chondrogenic differentiation assays on TDSCs (Fig. 4).

**Fig. 5:**
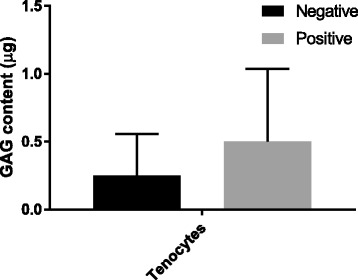
Total sulphated glycosaminoglycan (sGAG) content of cell pellets with (positive) or without (negative) chondrogenic induction. Error bars shown represent SD. Pairwise comparisons were performed using paired Student’s t-tests. sGAG content was not measured for TDSCs due to low cell numbers. *n* = 6 biological replicates

There was an increase in mean sGAG formation for tenocytes from 0.25 (±0.3) μg in negative samples to 0.5 (±0.54) μg in positive samples, however this was not significant. sGAG content was not analysed in TDSCs due to low cell numbers (Fig. 5).

**Fig. 6:**
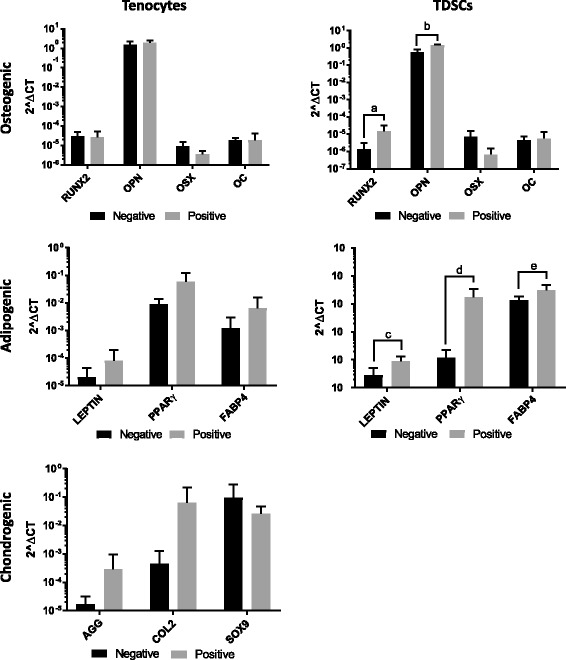
Gene expression analysis of lineage specific markers for murine tenocytes and TDSCs. Values are shown on a logarithmic scale and normalised to GAPDH. Error bars shown represent SD. Pairwise comparisons were performed using paired Student’s t-tests after Log_10_ transformation of data. ^a^*p* = 0.021, ^b^*p* = 0.02, ^c^*p* = 0.021, ^d^*p* = 0.021, ^e^*p* = 0.021. Chondrogenic marker genes are not shown for TDSCs due to low cell numbers. *n* = 6 biological replicates

Gene expression analysis of lineage specific genes showed a significant increase in the expression of osteogenic markers RUNX2 (runt-related transcription factor 2) and OPN (osteopontin) for TDSCs, however expression in tenocytes was similar between negative and positive samples (Fig. 6). There were small increases in all adipogenic marker genes, such as LEPTIN, FABP4 (fatty acid binding protein 4) and PPARγ (peroxisome proliferator-activated receptor gamma), for tenocytes, and much larger significant increases for TDSCs in positive samples compared to negative samples (Fig. 6). Similarly, there was an increase in the majority of chondrogenic markers, such as AGG (aggrecan) and COL2 (collagen type II) in positive samples compared with negative samples for tenocytes although these were not significant. Chondrogenic markers were not analysed in TDSCs due to low cell numbers (Fig. 6).

## Discussion

In this study we have isolated a population of cells in murine tendon that possess some of the traditional hallmarks of a stem cell: the ability to form colonies, the expression of stem cell markers and multipotency [39]. These findings are consistent with the published literature on murine TDSCs [12, 40–42]. The only discrepancy is the extended population doubling time observed in this study compared with previous reports. This could be explained by variations in cell isolation procedures. In this study we selected a low cell seeding density based on previous work in our group [15] and other studies [13, 16, 25–28], however some previous studies have used higher seeding densities. Alternatively, these differences may be due to mouse strain variation as research on murine mesenchymal stem cells (MSCs) has noted considerable variation in stem cell properties, including proliferation, between different strains of mice [43]. In addition, phenotypic differences of MSCs have been observed within certain strains of mice [44], highlighting the biological variation in murine stem cell populations. The TDSCs isolated in this study also stopped expanding at early passages which made certain assays impossible to perform due to low cell numbers. This may be due to stem cell quiescence, senescence or terminal differentiation and could indicate that these cells are not in fact stem cells but a progenitor cell population. For this reason we were unable to perform chondrogenic differentiation assays on TDSCs. We observed only moderate levels of chondrogenic differentiation for tenocytes which were low compared to reports in human tendon cells [45] and murine tendon tissue [46]. It is likely that the chondrogenic differentiation potential of TDSCs would be increased compared to tenocytes, as seen for osteogenic and adipogenic differentiation.

To our knowledge, no studies have compared the phenotype of murine tenocytes and TDSCs and we observed a number of phenotypic differences between these two cell populations. Tenocytes and TDSCs demonstrated different cell morphologies and colony forming ability as well as differences in the expression of certain stem cell markers, and some differences in multipotency. TDSCs generally conformed to the criteria of MSCs, as specified by the International Society for Cellular Therapy [39] (although chondrogenic potential could not be confirmed), whereas tenocytes did not due to a lack of adipogenic differentiation. The primary similarity between tenocytes and TDSCs was the expression of tenogenic markers such as tenascin C and thrombospondin 4, which was expected given that both cell populations were derived from tendon tissue. No studies have previously compared murine tenocytes and TDSCs, however such a comparison has been performed in other species [14, 15]. Our previous work demonstrated no discernible differences between tenocyte and TDSC populations in equine superficial digital flexor tendon, however a restricted differentiation potential was observed for equine TDSCs [15]. In contrast, a comparison of tenocytes and TDSCs in rabbit Achilles and patellar tendon demonstrated considerable differences in stemness between the two cell populations [14], which are more consistent with our study. The phenotypic differences observed in this study between tenocytes and TDSCs suggest that these cells are distinct populations with differing properties.

TDSCs have been used in a number of tissue engineering strategies to promote tendon healing with some encouraging results in human and animal models [16–20, 47, 48]. However, many of these studies do not state the exact TDSC isolation method used, or use varying cell seeding densities; in addition, many studies have not fully characterised the cells used for tendon repair. Therefore, it is possible that different tendon cell populations have been used across studies, which were not always defined as TDSCs. It is necessary to determine which tendon cell population is most effective for tendon repair. The increased stemness of murine TDSCs may promote tendon repair, however the poor proliferative potential of these cells is not conducive to tendon regeneration. Alternatively, murine tenocytes which demonstrated improved proliferative potential may provide a more suitable cell population for tendon regeneration. It is possible that the restricted differentiation potential of tenocytes may actually provide a therapeutic benefit during tendon healing by avoiding aberrant differentiation. Analysis of different tendon cell populations in human tendon has not yet been performed, however the presence of multiple tendon cell populations in several species [14, 15] would suggest the presence of more than one tendon cell population in human tendon. A comparison of tendon cell populations in humans is warranted, as well as investigation of the therapeutic potential of different tendon cell populations in vivo, which may highlight alternative, more effective tendon cell populations for human tendon repair strategies.

## Conclusion

In conclusion, we have isolated and characterised two distinct tendon cell populations from murine tail tendon with differential properties. These tendon cell populations may provide therapeutic benefit for tendon injury and determination of the most effective cell population for tendon regeneration strategies in both humans and animals requires further investigation.

## Abbreviations

AB: alcian blue
AGG: aggrecan
ALP: alkaline phosphatase
AR: alizarin red
CD: cluster of differentiation
cDNA: complementary deoxyribonucleic acid
COL2A1: collagen type II alpha 1
DMEM: Dulbecco’s modified Eagle’s medium
EDTA: ethylenediaminetetraacetic acid
FABP4: fatty acid binding protein 4
GAPDH: glyceraldehyde 3-phosphate dehydrogenase
IBMX: 3-isobutyl-1-methylxanthine
ITS: insulin transferrin selenium
MSC: mesenchymal stem cell
MKX: mohawk
OC: osteocalcin
OPN: osteopontin
ORO: oil red O
OSX: osterix
PDT: population doubling time
PPARγ: peroxisome proliferator-activated receptor gamma
qRT-PCR: quantitative real time-polymerase chain reaction
RNA: ribonucleic acid
RUNX2: runt-related transcription factor 2
Sca-1: stem cell antigen 1
SCX: scleraxis
SD: standard deviation
sGAG: sulphated glycosaminoglycan
SO: safranin O
TDSC: tendon-derived stem cell
TGFβ: transforming growth factor β
THBS4: thrombospondin 4
TNC: tenascin C
TNMD: tenomodulin
